# Temperature and Oxygen Dependent Metabolite Utilization by *Salmonella enterica* Serovars Derby and Mbandaka

**DOI:** 10.1371/journal.pone.0120450

**Published:** 2015-03-23

**Authors:** Matthew R. Hayward, Manal AbuOun, Martin J. Woodward, Vincent A. A. Jansen

**Affiliations:** 1 Department of Structural and Computational Biology, European Molecular Biology Laboratory, Meyerhofstraße 1, Heidelberg, 69117, Germany; 2 Department of Bacteriology, Animal Health and Veterinary Laboratories Agency, Woodham Lane, New Haw, Addlestone, Surrey, KT15 3NB, United Kingdom; 3 School of Biological Sciences, Royal Holloway University of London, Egham, Surrey, TW20 0EX, United Kingdom; 4 Department of Food and Nutritional Sciences, University of Reading, Whiteknights, Reading, RG6 6AP, United Kingdom; Indian Institute of Science, INDIA

## Abstract

*Salmonella enterica* is a zoonotic pathogen of clinical and veterinary significance, with over 2500 serovars. In previous work we compared two serovars displaying host associations inferred from isolation statistics. Here, to validate genome sequence data and to expand on the role of environmental metabolite constitution in host range determination we use a phenotypic microarray approach to assess the ability of these serovars to metabolise ~500 substrates at 25°C with oxygen (aerobic conditions) to represent the *ex vivo* environment and at 37°C with and without oxygen (aerobic/anaerobic conditions) to represent the *in vivo* environment. A total of 26 substrates elicited a significant difference in the rate of metabolism of which only one, D-galactonic acid-g-lactone, could be explained by the presence (*S*. Mbandaka) or the absence (*S*. Derby) of metabolic genes. We find that *S*. Mbandaka respires more efficiently at ambient temperatures and under aerobic conditions on 18 substrates including: glucosominic acid, saccharic acid, trehalose, fumaric acid, maltotriose, N-acetyl-D-glucosamine, N-acetyl-beta-D-mannosamine, fucose, L-serine and dihydroxy-acetone; whereas *S*. Derby is more metabolically competent anaerobically at 37°C for dipeptides, glutamine-glutamine, alanine-lysine, asparagine-glutamine and nitrogen sources glycine and nitrite. We conclude that the specific phenotype cannot be reliably predicted from the presence of metabolic genes directly relating to the metabolic pathways under study.

## Introduction

The non-typhoidal Salmonellas (NTS) colonise and may cause gastrointestinal disease in a wide range of vertebrates, including human and various livestock species [1]. Infection is often persistent, resulting in environmental contamination [2]. Veterinary, clinical and environmental isolates of *S*. *enterica* are routinely typed serologically for the somatic “O” antigen and the flagella “H” antigen, combinations of which allow the identification of over 2500 different serovars [3–6]. Some of these serovars are host specific, whereas others colonise a wide range of host species [7–11].

In previous work, we sequenced and compared the genomes of two frequently isolated serovars displaying host species preferences, *S*. Derby and *S*. Mbandaka [4–6,12]. In the UK these serovars show distinct isolation patterns, with *S*. Derby most frequently isolated from pigs (50%) and turkeys (40%) and *S*. Mbandaka most frequently isolated from cattle (65%) and chickens (20%) [5]. In addition, plant based feed is frequently identified as the route by which *S*. Mbandaka enters farms [9,13–15].

Commonly, the literature surrounding host association focuses on unique complements of virulence factors whilst environmental factors, such as the metabolic constitution of the surrounding environment are often overlooked [7,16,17]. Based on isolation data for *S*. Derby and *S*. Mbandaka the question arises as to whether bacterial metabolism contributes to the host and/or environmental isolation biases observed for these two serovars? Comparative genome analysis showed considerable genetic homology for metabolic functions between the two serovars [12]. Therefore, we hypothesised that there would be no difference between the two serovars in the metabolites they can utilise. To test this hypothesis we performed comparative phenotyping on one strain of each serovar which had been previously compared at the genome level, using Biolog (Biolog Inc., Hayward, USA) phenotypic microarrays [12]. We infer, from logistic models fit to respiratory readouts, differential utilisation of a wide range of small metabolites (comparative metabolic phenomics) under three experimental conditions: aerobic at 25°C to represent the *ex vivo* environment, aerobic and anaerobic at 37°C to represent *in vivo* environments.

We show that although the two serovars share a high degree of similarity in genes encoding metabolic functions, there are significant differences in small metabolite use which cannot be attributed to a difference in individual complements of metabolic genes. Due to the nature of the experimental design it was also possible to infer if utilisation of a given compound was temperature (used at 25°C or 37°C regardless of oxygen) or oxygen dependent (used at both temperatures only in the presence, or only at 37°C in the absence, of oxygen). This distinction contextualises the utilisation of compounds providing additional information that may contribute to developing testable hypotheses with regards to host/environmental adaptation. We relate these findings to the respective host species ranges of *S*. Derby and *S*. Mbandaka and conclude that functional genome annotation is, at least currently, a limited proxy for inferring metabolic phenotypes of *Salmonella*.

## Results

### Overview of the metabolic phenomes

In total, 26 test conditions showed a significant (p value < 0.01, n = 4) difference for at least one of three logistic respiratory curve parameters (duration of lag-phase prior to beginning to respire, log-phase rate of respiration and maximum respiratory response) between *S*. Derby D1 and *S*. Mbandaka M1 (Table 1). Of the test conditions that were significantly different, 18 were either for faster initial utilisation, a steeper gradient at log-phase or a greater degree of respiration by *S*. Mbandaka. There were no significant differences between *S*. Derby and *S*. Mbandaka for the utilisation of sulphur sources under any of the test conditions. Significant differences in nitrogen metabolism were only observed at 37°C under aerobic conditions. Significant differences in respiratory parameters for a given compound under one test condition were not observed under the other two test conditions. Of the observed differences five were found to be temperature dependent and nine dependent on the presence or absence of oxygen.

**Table 1:** Summary of significant differences in respiratory parameters between *S*. Derby and *S*. Mbandaka.

		25°C Aerobic			37°C Aerobic			37°C Anaerobic		
	Metabolite	μ	λ	A	μ	Λ	A	μ	λ	A
*Salmonella* Mbandaka	D-Galactonic acid-g-lactone (c)	M	M	M	-	-	-	-	-	-
	D-Glucosaminic Acid (c)	M	M	M	-	-	-	-	-	-
	D-Saccharic Acid (c)	M	+	+	+	+	+	+	+	+
	D-Trehalose (c)	M	+	+	+	+	+	+	+	+
	Fumaric Acid (c)	+	+	M	+	+	+	-	-	-
	Guanosine-2'-Monophosphate (p)	+	+	M	+	+	+	+	+	+
	Maltotriose (c)	M	+	+	+	+	+	+	+	+
	N-Acetyl-D-Glucosamine (c)	M	+	+	+	+	+	+	+	+
	N-Acetyl-β-D-Mannosamine (c)	+	+	M	+	+	+	-	-	-
	Succinic Acid (c)	+	+	M	+	+	+	-	-	-
	L-Serine (n)	+	+	+	+	M	+	+	+	+
	D-Fucose (c)	-	-	-	-	-	-	M	+	+
	Dihydroxy-Acetone (c)	-	-	-	-	-	-	+	M	+
	Thymidine-3'-Monophosphate (p)	-	-	-	+	+	+	+	+	M
	B-D-Allose (c)	-	-	-	-	-	-	M	+	+
*Salmonella* Derby	Glutamine-Glutamine (dP)	+	D	+	+	+	+	+	+	+
	Mucic Acid (c)	+	+	D	+	+	+	+	+	+
	Alanine-Lysine (dp)	-	-	-	D	+	+	-	-	-
	Asparagine-Glutamine (dp)	+	+	+	+	+	D	+	+	+
	Glycine (n)	-	-	-	D	+	+	+	+	+
	Nitrite (n)	-	-	-	+	+	D	+	+	+
	Glycylalanine (dp)	+	+	+	+	+	+	+	D	+
Both	D-Tagatose (c)	-	-	-	-	-	-	M	+	D
	D-Melibiose (c)	M	D	+	+	+	+	+	+	+
	Mono-Methyl Succinate (c)	+	D	M	+	+	+	-	-	-

The parameters μ (duration of lag-phase), λ (log-phase gradient) and A (maximum dye reading) parameters fit to logistic respiratory curves between *S*. Derby D1 and *S*. Mbandaka at 25°C and 37°C aerobic and anaerobic. The symbols (D) and (M) signify respiratory parameters which under competitive conditions would favour *S*. Derby D1 and *S*. Mbandaka M1 respectively. The symbol (+) signifies a logistic respiratory curve for both *S*. Derby D1 and *S*. Mbandaka M1 with parameters that were not significantly different and the symbol (-) signifies no respiratory response from either strain. The nature of the test metabolite in the media formulation are denoted by (c) carbon, (p) phosphorous, (n) nitrogen and (dp) dipeptide.

### Differences in metabolite utilisation between *S*. Derby and *S*. Mbandaka incubated aerobically at 25°C

At 25°C under aerobic conditions a total of 14 of the 26 significantly different respiratory kinetic parameters were observed, and of these 11 showed a faster initial utilisation or greater degree of respiration for *S*. Mbandaka. Under these conditions there were no compounds *S*. Derby could utilise that *S*. Mbandaka could not.

**Fig 1:**
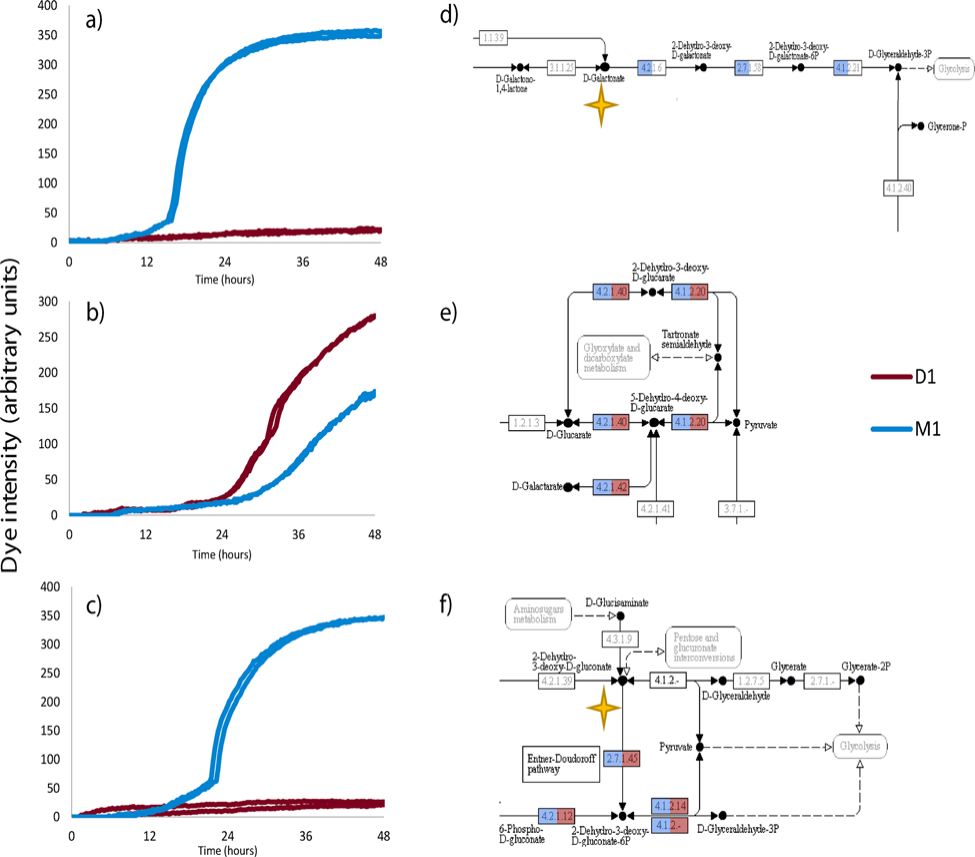
Relationship between respiratory response and metabolic pathways of S. Derby and S. Mbandaka. Respiratory responses which distinguish *S*. Derby and *S*. Mbandaka under aerobic conditions at 25°C and the corresponding metabolic pathway for the metabolism of the compound from uptake to glycolysis. Only *S*. Mbandaka respires on D-galactonic acid-g-lactone (DGL) (a), this can be attributed to the lack of pathway genes (e) (KEGG map 00052, created 31/5/12). Likewise, *S*. Derby is unable to respire on D-glucosaminic acid (DGA) while *S*. Mbandaka can (c) yet both serovars possess the same pathway genes (g) (KEGG map 00030, created 9/3/13). For both DGL and DGA there were gaps in the SEEDmodel reconstruction which prevented the metabolism of the compounds, these were filled using ECBLAST and SEEDviewer BLASTp (yellow stars). Both serovars contain the same pathway genes for the metabolism of mucic acid (f) (KEGG map 00053, created 28/9/09) yet the area under the respiratory curve for this compound, and not the log phase gradient or lag phase, was significantly greater for *S*. Derby (b).

D-galactonic acid-g-lactone (DGL) was one of two compounds on which *S*. Mbandaka could respire and *S*. Derby could not (Fig. 1a). *S*. Mbandaka respired on DGL at 25°C but not at 37°C either aerobically or anaerobically. Hence utilisation of this compound was temperature dependent. *S*. Derby lacked the *dgo* operon which allows the passage of D-galactonic acid from the external environment in to glycolysis (Fig. 1d). BLASTp showed that both serovars contained genes which share 71% sequence identity with the same portions of a gene encoding a Gluconolactonase (EC3.1.1.25) which converts D-galactonic acid-g-lactone in to D-galactonic acid, allowing the utilisation of the remainder of the pathway encoded by the *dgo* operon of *S*. Mbandaka, and hence represents a metabolic dead-end for *S*. Derby.

The second compound that *S*. Mbandaka could utilise that *S*. Derby could not was D-glucosaminic acid (Fig. 1c). Neither serovar elicited a respiratory response to D-glucosaminic acid at either 37°C aerobically or anaerobically. Therefore utilisation of this compound was temperature dependent. Surprisingly, both serovars contain the same metabolic genes for the secondary metabolism of D-glucosaminic acid and its subsequent passage into glycoloysis (Fig. 1d).

The respiratory responses to the carbon sources N-acetyl-D-glucosamine, D-saccharic acid, D-trehalose and maltotriose were significantly faster for *S*. Mbandaka with log phase slopes between 1.30 and 2.23 times steeper than that of *S*. Derby. *S*. Mbandaka achieved a greater degree of respiration on the carbon sources succinic acid, fumaric acid and N-acetyl-beta-D-mannosamine with 1.25 to 4.55 fold that of *S*. Derby. Utilisation of carbon sources succinic acid, fumaric acid and N-acetyl-beta-D-mannosamine were oxygen dependent since both serovars respired on these compounds at 37°C, under aerobic but not anaerobic conditions.

*S*. Mbandaka achieved a greater degree of respiration (1.5 times greater) than *S*. Derby on the phosphate source guanosine-2’-3’-cyclic monophosphate. Both serovars showed a positive respiratory kinetic at 37°C aerobically and anaerobically.

Initial utilisation of D-melibiose after *S*. Mbandaka inoculation took 1.76 times longer than *S*. Derby, but once started it achieved a log-phase gradient 1.76 times steeper. Both serovars respired on D-melibiose at 37°C aerobically and anaerobically. *S*. Mbandaka was 1.33 times slower than *S*. Derby at initiating the use of mono-methyl-succinate, but achieved a maximum respiratory response 1.54 times greater. Therefore, utilisation of mono-methyl-succinate was oxygen dependent.

The only carbon source that elicited a greater respiratory response from *S*. Derby at 25°C was mucic (galactaric) acid. On this compound *S*. Derby reduced 1.64 times more dye than *S*. Mbandaka (Fig. 1b). Both serovars respired on mucic acid at 37°C under aerobic and anaerobic conditions. Both serovars contain the same genes for metabolism of mucic acid (Fig. 1e).

### Differences in metabolite utilisation between *S*. Derby and *S*. Mbandaka incubated aerobically at 37°C

At 37°C *S*. Derby achieved 3.44 times the maximum dye reduction of *S*. Mbandaka when utilising the nitrogen source nitrite. Nitrite metabolism was found to be temperature dependent since both serovars also respire on the metabolite at 37°C anaerobically and not at 25°C aerobically. *S*. Derby had a lag-phase 2.82 times shorter on glycine than *S*. Mbandaka. Utilisation of glycine was also found to be temperature dependent. The lag-phase of *S*. Derby on L-serine was 1.93 times shorter than *S*. Mbandaka, both respired on the metabolite at 25°C aerobically and 37°C anaerobically. *S*. Derby had a log-phase gradient 2.78 times steeper on alanine-lysine dipeptides than *S*. Mbandaka. Neither respired on alanine-lysine dipeptides at 25°C aerobically or 37°C anaerobically. *S*. Derby was able to achieve a maximum dye reduction 1.14 times greater than *S*. Mbandaka on asparagine-glutamine dipeptides. Both serovars respired on asparagine-glutamine dipeptides at 25°C and 37°C under aerobic conditions.

### Differences in metabolite utilisation between *S*. Derby and *S*. Mbandaka incubated anaerobically at 37°C

*S*. Derby and *S*. Mbandaka differ significantly on the utilisation of four carbon sources when grown anaerobically at 37°C. *S*. Derby lags behind *S*. Mbandaka in the utilisation of dihydroxy-acetone with a lag-phase 1.61 times longer. For D-tagatose, *S*. Mbandaka began to utilise this compound with a log-phase gradient 2.26 times steeper than that of *S*. Derby. However, *S*. Derby achieved 1.05 times the maximum dye reduction of *S*. Mbandaka. Neither serovar respired on dihydroxy-acetone, or D-tagatose at 25°C or 37°C aerobically. The lag-phase of *S*. Derby when utilising the dipeptide glycylalanine was nine times shorter than that of *S*. Mbandaka. Both serovars respired on glycylalanine at 25°C and 37°C aerobically.

## Discussion

In this study we profiled the metabolic phenome of two *S*. *enterica* serovars, Derby and Mbandaka. The data generated validated the one predicted metabolic difference derived from genome sequence data namely that *S*. Mbandaka can utilise DGL whereas *S*. Derby cannot, correlating with the presence and absence of the *dgo* operon respectively. This adds to previous work by Anjum et al. (2005) and Pan et al. (2009) who demonstrated phenotypic differences associated with gene presence or absence in the evolution of *S*. Enteritidis phage types [18,19]. However, of importance is the observation that it is not always possible to infer isotypic phenotypes from sequencing data of different organisms even though they share a high degree of genetic homology for the encoding genes.

The parameters used represent three biologically distinct phases of bacterial response to the utilisation and depletion of available metabolites. In a closed system, these parameters are influenced by gene regulation, metabolic rate, resource abundance and toxic by-product build up [20,21]. Respiration may occur independently of growth, though the logistic respiratory dynamics of *S*. *enterica* in the well of a phenotypic microarray probably reflects logistic growth dynamics. Conversely, a linear respiratory dynamic would most likely represent a stable population density and a constant rate of metabolism, as respiration is measured cumulatively through the reduction of a tetrazolium dye by NADH. This dynamic could be seen when metabolic flux does not lead to biomass incorporation or where the media, temperature or the presence or absence of oxygen have a bacteriostatic effect on the culture. In the absence of respiration, no cellular division can occur [22]. Under these assumptions the conclusions drawn from the logistic respiratory kinetics observed here may be extrapolated to the ability of the bacteria to replicate on these metabolites when provided as a sole source of a nutrient.

We showed that only *S*. Mbandaka M1 could respire on DGL, as anticipated from genome sequence data, yet unexpectedly only in a temperature dependent manner (Table 1). Comparative functional genomics also identified several other incomplete metabolic pathways in either *S*. Derby D1 or *S*. Mbandaka M1 that were complete in the other [12]. None of these differences in metabolic pathways manifest here as differences in proficiency of metabolism. There were approximately 251 hypothetical genes that possessed less than 90% bidirectional amino acid sequence homology distinguishing the chromosome sequences of *S*. Derby D1 and *S*. Mbandaka M1 [12]. These genes have no putative function and therefore could be metabolic genes with distinct functions that may explain the unforeseen metabolic differences described here. Gene expression studies may also identify which genes relating to transport, primary and secondary metabolite reactions, correlate with the differences observed. The converse of this observation is the inability of *S*. Derby to respire on D-glucosaminic acid under any of the test conditions, while *S*. Mbandaka was able to utilise it at 25°C, in spite of the presence of a full pathway from transport to glycolysis in both serovars (Fig. 1c and g).

Previous studies have found an association between *S*. Mbandaka and soybean based feed [9,13–15]. Interestingly, the metabolites *S*. Mbandaka performed better on at 25°C compared to *S*. Derby (D-saccharic acid, succinic acid, D-trehalose, D-mellibiose, mono-methyl succinate and fumaric acid) are all components of the soybean metabolome (SoyMetDB accessed 9/6/13) although these sugars are readily utilised by, for example, a porcine host (KEGG maps for *Sus scrofa*: 00020 created 31/5/12, 00052 created 31/5/12, 00053 created 30/7/12, 00500 created 9/7/12, 00520 created 25/1/13) [5,23–25]. Hence, it may be posited that these metabolic competences may confer competitive advantage under appropriate environments and conditions, i.e. aerobic conditions at 25°C such as in animal feed, *ex vivo*, prior to ingestion and metabolism by the host.

In this study, lag-phase was typically much longer than log-phase (data not shown). In the case of a metabolite such as glycylalanine, incubated at 37°C anaerobically, *S*. Derby reached plateau phase of respiratory dynamics before *S*. Mbandaka had started to respire, most likely due to the time taken for changes to occur in gene expression though this would require experimental verification. Interestingly, both serovars were able to use glycylalanine at 25°C and 37°C aerobically without any significant differences in respiratory parameters (μ, λ and A); this may suggest that *S*. Derby is better adapted, by being quicker at utilising this metabolite when in an anoxic environment. Glycine elicited a much steeper log-phase gradient in *S*. Derby than *S*. Mbandaka when incubated at 37°C aerobically. Glycine is used as a sweetener and pH buffer in animal feed, and therefore may be in high abundance in the gastro-intestinal tract (GIT) [26]. This further strengthens observations made in previous studies that *S*. Derby is better adapted to the porcine GIT then *S*. Mbandaka. In particular the observation that *S*. Derby colonises porcine derived cells faster than *S*. Mbandaka. This difference correlates with the expression of the newly identified *Salmonella* pathogenicity island, SPI-23. Furthermore, mutation of the gene *potR* found on SPI-23, a gene which is highly expressed in the porcine jejunum compared to the colon, was essential for surface pili detachment, adhesion and invasion of porcine tissue [27].

Longitudinal studies of the proportion of different serovars of *Salmonella* in livestock as they progress found that *S*. Mbandaka decreased in prevalence [28,29]. *S*. Derby, on the other hand, was shown to have a low prevalence in nursery stage pigs, becoming the dominant serovar by finishing stage [29,30]. It is possible that the metabolic phenomic differences described here, amongst many other environmental and host derived factors, may contribute to a difference in host occupation, a hypothesis that could be tested, for instance, by using longitudinal mixed culture inoculation studies of *S*. Derby and *S*. Mbandaka in different environments and hosts.

The conditions used reflect aspects of the *ex vivo* and *in vivo* environments in which these organisms are found and may reflect the biology of the two serovars [31]. Thus, the conclusions that can be drawn from comparative genomic studies alone may prove troublesome and we argue that the approach used here can be expanded to additional environmental stimuli and generally applied to *Salmonella* serovars to give additional insights into their phenotypic diversity, over and above those gained by genome sequence analysis alone. That said, the next step in developing these concepts is the need to relate phenotype to the specific sequence differences in genes and regulatory regions in isofunctional gene sets in the different serovars and also undertake transcriptional studies including analyses of small regulatory RNA species [32].

## Methods and Materials

### Determining the metabolic phenome of *S*. Derby and *S*. Mbandaka

Analysis of the metabolic phenome of *S*. Derby and *S*. Mbandaka using phenotypic microarray technology was performed as previously described for aerobic and anaerobic conditions but with the inclusion of assays for different temperatures [33,34]. In brief, phenotypic microarray plates, PM1, PM2A, PM3B, PM4A and PM6, containing different sources of carbon, nitrogen, phosphorous, sulphur and dipeptides, were purchased from Biolog Inc, Hayward CA. Experiments were carried out in duplicate, on two separate occasions. Genome sequenced isolates *S*. Derby D1 originally isolated from pig and *S*. Mbandaka M1 originally isolated from cattle, were grown on LB agar plates incubated at 25°C aerobically, 37°C aerobically and 37°C anaerobically for 16 hours [12]. All subsequent steps were performed either in an anaerobic cabinet or on a bench top so as to maintain preconditioning to the oxygen concentration of the test environment. For the anaerobic plates all reagents were incubated for 24 hours with loose lids, in the anaerobic cabinet, to allow oxygen to diffuse out of solution. Colonies were re-suspended in M9 minimal medium without glucose for PM1 and PM2A (carbon sources) and PM6 (dipeptides) and a reduced M9 salts lacking nitrogen (PM3B nitrogen sources), sulphur (PM4A wells A1 to E12 sulphur sources) and phosphate (PM4A wells F1 to H12 phosphorous sources) to 85% turbidity against a Biolog standard [35]. Wells of the PM plates were inoculated with 100μl of bacterial suspension. Plates PM3B and PM4A were supplemented with 8mg/ml D-gluconic acid sodium salt as a carbon source. The inoculation solution was also supplemented with 3μg/ml thiamine. Anaerobic plates were sealed in to 4oz Whirl-PakW Long-Term Sample Retention Bags (Nasco) with an AGELESSW oxygen absorber and a CO2GEN compact sachet (Oxoid); the end of the bag was heat sealed prior to removal from the anaerobic cabinet. Phenotypic microarrays were read at 15 minute intervals for 72 hours during incubation in an Omnilog incubator at either 25°C or 37°C reflecting initial culturing and preparation conditions.

### Analysis of Biolog phenotypic microarrays

Results were exported using Omnilog-PM Kinetics software v1.3 (Biolog). All subsequent analysis was performed using R statistical language v2.15.2 utilising the packages OPM v0.8 and Grofit v1.1 [36–38]. The respiratory response for each metabolite of each repeat was zeroed to the value of the first measurement to ensure comparability between repeats and treatments. A separate spline model was fitted to the respiratory response, using the do_aggr() function from the OPM package, for each well under each condition. The models were fitted to the first 48 hours of reads for the 25°C aerobic and 37°C anaerobic plates, and for the first 24 hours for the 37°C aerobic plates because by these time points a respiratory response was observed if one was to be observed over the 72 hour incubation period. This was done for practical reasons, as it greatly reduced the time taken to fit the models. The model parameters μ (log-phase gradient), λ (the duration of lag-phase) and A (the maximum reading over the duration of the assay), were extracted for further analysis. Significant differences between the serovars were identified through performance of a two-tailed Student's t-test with a p-value of 0.01 or lower. Under conditions showing no significant differences between strains a positive respiratory response was identified when a logistic curve could be fitted. A flat line signified no respiratory response.

### Metabolic network gap filling and analysis

Metabolic network reconstructions were produced and analysed using modelSEED [12]. Two gaps in the metabolic networks which prevented the theoretical passage of D-galactonic acid-g-lactone and D-glucosaminic acid from the external environment in to glycolysis were filled by first identifying genes which corresponded to the EC numbers EC3.1.1.23 and EC4.3.1.9 respectively using ECBLAST, then by confirming the presence of these genes in the genomes of *S*. Derby D1 (RAST_ID: 28144.16) and *S*. Mbandaka M1 (RAST_ID: 192954.16) using SEEDviewer BLASTp function.
